# Effects of Breed and Stage of Lactation on Milk Fatty Acid Composition of Italian Goat Breeds

**DOI:** 10.3390/ani9100764

**Published:** 2019-10-03

**Authors:** Sarah Currò, Carmen L. Manuelian, Massimo De Marchi, Salvatore Claps, Domenico Rufrano, Gianluca Neglia

**Affiliations:** 1 Department of Agronomy, Food, Natural resources, Animals and Environment, University of Padova, 35020 Legnaro (PD), Italy; sarah.curro@phd.unipd.it (S.C.); carmenloreto.manuelianfuste@unipd.it (C.L.M.); 2 Council for Agricultural Research and Agricultural Economy Analysis—Research Centre for Animal Production and Aquaculture, 85051 Bella Muro (PZ), Italy; salvatore.claps@crea.gov.it (S.C.); drufrano@tiscali.it (D.R.); 3 Department of Veterinary Medicine and Animal Production (DMVPA), University of Naples Federico II, Naples, 80137 Napoli, Italy; neglia@unina.it

**Keywords:** atherogenicity index, conjugated linoleic acids, desaturation index, local goat breeds, fatty acids

## Abstract

**Simple Summary:**

Milk fatty acid composition affects human health and dairy products flavor. In particular, some saturated fatty acids increase the risk of cardiovascular diseases, whereas conjugated linoleic acid inhibits carcinogenesis and reduces atherosclerosis and diabetes. Moreover, a greater amount of some short chain fatty acids increase the goaty flavor of dairy products. The objective of this study was to evaluate the breed and week of lactation effects on milk fatty acid profile of 5 Italian local goat breeds and a cosmopolitan breed reared in the same farm. Results showed that the fatty acid profile was mainly affected by the week of lactation. Saturated fatty acids were abundant in early lactation and unsaturated fatty acids were abundant in late lactation. Local goat breeds produced milk with lower concentration of saturated fatty acids than the cosmopolitan breed. This study may contribute to valorize milk of Italian local goat breeds which seems to have a healthier profile than milk of the cosmopolitan breed.

**Abstract:**

Fatty acid (FA) profile plays an important role on human health and on sensory quality of dairy products. There is few information about breed influence on milk FA profile of local goat breeds. This study aimed to characterize and compare the milk FA profile of 5 local endangered goat breeds (Garganica, Girgentana, Jonica, Maltese, and Mediterranean Red) and a cosmopolitan breed (Saanen) reared in the same farm during a complete lactation. A total of 252 milk samples were collected monthly from 42 goats (7 goats per breed) and analyzed for gross composition and FA profile. Individual FA was determined using gas-chromatography. Data were analyzed using a mixed model with repeated measures with breed and week of lactation as fixed effects. Results showed that the FA profile was significantly affected by week of lactation and only few FA by breed effect (*p* < 0.05). Overall, the main differences were found between Saanen and local breeds. This study contributed to the characterization of goat milk FA profile, and it may be of interest for the valorization of milk from local goat breeds which seem to have a healthier profile than milk of the cosmopolitan breed.

## 1. Introduction

From 2007 to 2017, goat milk production in Europe increased by 5% [1]. In 2017, goat milk production represented 1.24% of the total European milk production (227 × 10^6^ t), with France (21%), Greece (20%), and Spain (17%) being the main producers [1]. In Europe, 95% of goat milk is transformed into cheese [2]. The increase of goat milk production reflects the consumer’s trend towards an increased intake of goat milk and dairy products. Generally, milk shows a neutral effect on cardiovascular health, whereas fermented milk and cheese may have also a positive effect; indeed, the fermented dairy food consumption increases the intestinal microbiota, reduces the low density lipoprotein cholesterol, hypertension risk, and cardiovascular diseases [3]. Goat milk is richer in unsaturated fatty acids (UFA), short chain fatty acids (SCFA), and medium chain fatty acids (MCFA) than cow milk [4,5], and thus, it is of great interest for nutritional and beneficial aspects in human diet and health [3]. Goat dairy products consumption provides some antithrombotic effects and a potential reduction in platelet aggregation [3]. Moreover, it is easier to digest due to the smaller fat globule dimension (3.19 to 3.50 µm) [4,5,6] and it is less allergenic because of the lower amount of α_S1_ casein than cow milk [6].

Fatty acids (FA) play an important role in human health [7,8]. It has been reported that polyunsaturated FA (PUFA) protect against heart attack risk, improve brain function, and reduce risk of dementia [8], and conjugated linoleic acids (CLA) inhibit the carcinogenesis and reduce the risk of atherosclerosis and diabetes [9,10]. Recently, the negative role attributed to saturated fatty acids on human health was re-examined [3]; indeed, not all individual SFA have a negative impact on human health. In particular, SCFA and MCFA (composed mainly of SFA) are a source of direct energy available for the enterocytes, whereas long chain fatty acids (LCFA) are stored in adipose tissues [11,12]. About 60% of milk FA derive from the mobilization of adipose tissue or from feed intake, and the remaining 40% are synthesized de novo by the mammary gland using the acetate and β-hydroxybutyrate from the rumen as substrates [13,14,15]. De novo FA are from C4:0 to C14:0, 50% of C16:0 and all odd FA, whereas 50% of C16:0 and all FA from C18:0 onwards derive from the arterial blood [13]. Moreover, the mammary gland shows an important Δ-9 desaturase activity which adds a double bond in the cis Δ-9 position converting C14:0 into C14:1, C16:0 into C16:1, and C18:0 into C18:1 and C18:2 [13]. 

Milk FA composition is mainly affected by feeding [16,17]; however it is difficult to evaluate the effect of feeding and stage of lactation separately [18], being feeding closely connected to the season and lactation stage [19]. Overall, diets richer in concentrate than forage cause the reduction of the ruminal pH and the activity of cellulolytic bacteria; this leads to the increase of trans-10, cis-12 CLA synthesis in milk which could induce milk fat depression syndrome [16,17]. Milk FA profile is affected also by breed and lactation stage. For example, Strzalkowska et al. [20] reported differences of FA profile in early, mid, and late lactation of Polish White Improved goats. Yurchenko et al. [21] demonstrated that milk from Saanen and Landrance goat breeds fed the same ration differed from C4:0 to C14:0, C16:0, C16:1, and C18:1. However, the existing literature about FA profile characterization is limited to a restricted number of breeds and to our knowledge few studies have investigated the milk FA composition of older, endangered goat breeds. 

Although the endemic breeds are well adapted to the environmental conditions showing a greater rusticity and resistance to diseases than cosmopolitan breeds [22,23], they have undergone a massive substitution with more yielding breeds [24]. Among European countries, Italy has the greatest number of local goat breeds (55), from which 61% are at risk of extinction, 10% are not at risk, and the risk status of 29% of the breeds is unknown [25]. To maintain the variability among breeds it is important to ensure conservation of livestock genetic resources as part of socio-cultural and livestock heritage of the country [23,26]. Recent studies have investigated milk [27,28,29] and dairy products [29,30] gross composition and characterized the casein haplotypes [31] and minerals [32] on some Italian local breeds. The aim of the present study was to characterize and compare milk FA composition of 5 local endangered goat breeds of South Italy (Garganica, Girgentana, Jonica, Maltese, and Mediterranean Red) and a cosmopolitan dairy specialized goat breed (Saanen) during a complete lactation under the same farm conditions.

## 2. Materials and Methods

### 2.1. Animals and Management Conditions

The study was conducted from March to August 2016 in the experimental farm of the Council for Agricultural Research and Agricultural Economy Analysis (CREA, Potenza, Italy). Experimental procedures and animal care conditions followed the recommendations of European Union directive 86/609/EEC. A general description of the breeds used in the study can be retrieved from Currò et al. [27]. Forty-two dairy goats of 6 breeds (7 does per breed; Garganica, GA; Girgentana, GI; Jonica, JO; Maltese, MA; Mediterranean Red, MR; and Saanen, SA) of parity order from 1 to 5, similar body condition score (between 2.5 and 3.0; 1 = very thin to 5 = very fat, with 0.5 point-increment [33]) and body weight at the beginning of lactation of 47 ± 3 kg for GA, 44 ± 5 kg for GI, 45 ± 5 kg for JO, 45 ± 5 kg for MA, 47 ± 3 kg for MR, and 61 ± 6 kg for SA were enrolled in the study. All does were under the same managerial conditions and kidded twins in February. Kids were kept with their mothers until 40 days of age but, during that period, dams were separated from their kids 24 h prior to sampling. Does were milked twice a day (morning and evening) in a double 24-stall herringbone low-line milk pipeline milking parlor (Alfa Laval Agri; Monza, Italy) equipped with recording jars and electronic pulsators at a vacuum of 38 kPa, 90 pulses/min, and 60% pulsation ratio. The pre-milking phase consisted of forestripping only, without any preparation of udder and teats.

During the whole study, does grazed together 8 h/day in a natural pasture and were supplemented ad libitum with polyphite hay (60%–65% of grasses, mainly Avena sativa L. and 35–40% of legumes, mainly Vicia sativa L.) in the shelter. Moreover, goats received a commercial concentrate in the milking parlor composed of maize, wheat bran and flour, maize flour, sunflower germ flour, sugar beet molasses, soybean meal (48% crude protein), calcium carbonate, sodium chloride, sodium bicarbonate, I (5 mg/kg), Mn (50 mg/kg), and Zn (125 mg/kg). The chemical composition and nutritional value of the hay and the concentrate are reported in Table 1. The amount of concentrate administered to the animals was adjusted every 15 days throughout lactation considering mean body weight and mean milk production per breed according to NRC (2007) requirements [34]. During the whole study, the amount of concentrate administration ranged from 0.6 to 1.0 kg/day for local breeds and 0.9 to 1.3 kg/day for SA breed, being the greatest amount at the beginning and the lowest at the end of lactation.

### 2.2. Sample Collection and Chemical Analysis

From 4 to 24 weeks of lactation, individual milk yield (kg/day) was recorded using the recording jars in the milking parlor as the sum of morning and evening milkings. In addition, individual milk samples (50 mL; n = 252) were collected and divided in two aliquots. One aliquot was stored at 4 °C and transferred to the milk laboratory of the Breeders Association of Basilicata region (Potenza, Italy) for the determination of fat, protein, and lactose percentages using MilkoScan FT6000 (Foss Electric, Hillerød, Denmark). Fat-corrected milk at 3.5% (FCM 3.5%) was estimated according to Pulina et al. [35]:

FCM 3.5% = milk yield × (0.634 + 0.1046 × fat%)(1)

Somatic cell count (SCC; cells/mL) was determined using Fossomatic FC (Foss Electric, Hillerød, Denmark) and transformed to somatic cell score (SCS) through the formula [36]:

SCS = 3 + log_2_(SCC/100,000) (2)

The second aliquot was stored at −80 °C and transferred to the laboratory of the Department of Agronomy, Food, Natural Resources, Animals and Environment of the University of Padova (Legnaro, Italy) for the FA profile analysis. Milk fat was extracted from a milk subsample (5 mL) following the accelerated solvent extraction method by ASE 200 (Dionex Corp., Sunnyvale, CA, USA). Samples were put in 22 mL stainless steel extraction cells for fat extraction using hexane:isopropanol (2:1) as solvent. After the solvent evaporation, 40 mg of fat was transferred into tubes for the FA transesterification and methylation following an internal method adapted from Christie [37]. Transesterification and methylation processes were performed adding 2 mL of normal hexane and 100 μL of sodium methylate 1 M in the tube in which were contained 40 mg of fat. After 20 min of reaction time in a stirrer, 150 μL of oxalic acid in ethyl ether and 4 mL of sodium sulphate (0.47 M) were added in the tube. Successively, fatty acids methyl esters solution was centrifuged at 693 × g for 10 min at 10 °C. The FA gas-chromatographic analysis was performed using an Agilent 7820A GC System (Agilent Technologies, Santa Clara, CA, USA) equipped with an automatic sampler G4567A (Agilent Technologies) and flame-ionization detector. An Omegawax capillary GC column (24,136 Supelco; Sigma-Aldrich, Castle Hill, Australia), with a long of 30 m, inner diameter of 0.25 mm, and film thickness 0.25 μm, was used. Hydrogen was used as a carrier gas at a constant flow rate at 100 °C with an average speed of 30 cm/s. A split injection sleeve was used. The injector and detector temperature was set at 250 °C. The initial temperature of oven was at 50 °C for 2 min, and then increased from 4 °C/min to 220 °C and held for 18 min. Each individual FA was identified by comparing its retention time with that of a standard FA (FAME mix C4-C24 #18919-1AMP and octadecadienoic acid conjugated methyl ester; Supelco, Sigma-Aldrich). Individual FAs were calculated using GC/MSD ChemStation Software (Agilent Technologies) and expressed as percentage of total identified FA.

Identified individual FA were grouped in the following relevant FA groups: C14:0, which included C14:0 and C14:0 iso form; C15:0, which included C15:0, and C15:0 iso and anteiso forms; C16:0, which included C16:0 and C16:0 iso form; C17:0, which included C17:0, and C17:0 iso and anteiso forms; C18:0, which included C18:0 and C18:0 iso form; C18:1 which included C18:1n7 and C18:1n9; C18:2n6 which included C18:2n6 and C18:2n6 trans; n3, which included C18:3n3, C18:4n3, and C20:5n3; n6, which included C18:2n6, C18:2n6 trans, C18:3n6, C20:2n6, C20:3n6, C20:4n6, and C22:2n6; CLA, which included geometric isomers of C18:2n6; SFA, which included C4:0, C6:0, C7:0, C8:0, C10:0, C11:0, C12:0, C13:0, and C13:0 iso form, C14:0 and C14:0 iso form, C15:0, and C15:0 iso and anteiso forms, C16:0 and C16:0 iso form, C17:0, and C17:0 iso and anteiso forms, C18:0, and C18:0 iso and anteiso forms, C19:0, C20:0, C21:0, C22:0, C23:0, and C24:0; monounsaturated FA (MUFA), which included C12:1, C14:1n7, C15:1, C16:1n9, C16:1n7, C16:1, C17:1n7, C18:1n9, C18:1n7 C20:1n9, and C22:1n9; PUFA, which included C18:2n6, C18:2n6 trans, C18:2 (and isomers), C18:3n6, C18:3n3, C18:4n3, C20:2n6, C20:3n6, C20:4n6, C20:5n3, and C22:2n6; UFA, which was the sum of MUFA and PUFA; CLA, which included geometric isomers of C18:2n6; SCFA, which included C4:0, C6:0, C8:0, and C10:0; MCFA, which included C11:0, C12:0, C12:1cis, C12:1trans, C13:0, and C13:0 iso form, C14:0 and C14:0 iso form, C14:1n7, C15:0, and C15:0 iso and anteiso forms, C15:1trans, C16:0, and C16:0 iso form, C16:1n7, C16:1n9, and C16:1 trans; LCFA, which included C17:0, and C17:0 iso and anteiso forms, C17:1, C18:0, and C18:0 iso form, C18:1n9, C18:1n7cis, C18:2n6, C18:2n6 trans, C18:3n6, C19:0, C18:3n3, C18:4n3, C20:2n6, C20:3n6, C20:4n6, C20:5n3, and C22:2n6; desaturation index of C16:0 (DI C16:0), calculated as (C16:1n7 + C16:1n9 + C16:1trans)/(C16:0 + C16:0:iso + C16:1n7 + C16:1n9 + C16:1t) × 100; desaturation index of C18:0 (DI C18:0), calculated as (C18:1n9 + C18:1n7cis)/(C18:0 + C18:0:iso + C18:1n9 + C18:1n7cis) × 100; atherogenicity index (AI), calculated as (C12:0 × (4 × C14:0) + C16:0)/UFA; elongation index (EI), calculated as (C8:0 + C10:0 + C12:0 + C14:0)/(C4:0 + C6:0); and thrombogenic index (TI), calculated as (C14:0 + C16:0 + C18:0)/[(0.5MUFA) + (0.5 n6) + (3 × n3) + (n3/n6)].

### 2.3. Statistical Analysis

According to fat to protein ratio (F/P), which is considered an indicator of metabolic status of goats [38,39], milk samples with F/P < 0.94 were discarded prior to statistical analysis. Normal distribution of the residuals for each trait was assessed. The complete record from samples presenting outliers for major FA and groups of FA were deleted, whereas outliers for minor FA were treated as missing values. Sources of variation of milk yield, FCM 3.5%, SCS, milk composition and individual, groups, and indices of FA were investigated using the MIXED procedure of SAS v9.4 (SAS Inst. Inc., Cary, NC, USA) with repeated measures according to the following mixed linear model:

y_ijk_ = µ + Breed_i_ + Week_j_ + (Breed × Week)_ij_ + Goat_k_(Breed_i_) + Ɛ_ijk_ (3)

where y_ijk_ is the dependent variable (milk yield, FCM 3.5%, SCS, fat percentage, protein percentage, lactose percentage or each individual, group, or index of FA); µ is the overall intercept of the model; Breed_i_ is the fixed effect of the *i*th breed (*i* = GA, GI, JO, MA, MR, SA); Week_j_ is the fixed effect of the *j*th week of lactation (*j* = 1 to 6, corresponding to every 4-week sampling); (Breed × Week)_ij_ is the fixed interaction effect between breed and week of lactation; Goat_k_(Breed_i_) is the random effect of the *k*th goat nested within the *i*th breed ~N(0, σ^2^_Goat (Breed)_); and Ɛ*_ijk_* is the random residual ~N(0, σ^2^_Ɛ_). In a preliminary analysis, the interactions Parity × Week of lactation and Breed × Parity were not significant and thus they were removed from the final model. Multiple comparisons of least squares means (LSM) were performed for the main effects of breed and week of lactation using Tukey’s test adjustment. Significance was set at *p* < 0.05.

## 3. Results

### 3.1. Breed Effect

The analysis of variance indicated that breed affected (*p* < 0.05) all studied traits except for fat percentage (Table 2). The greatest milk yield (1.73 kg/day) and FCM 3.5% (1.67 kg/day) were observed for SA, and the lowest for GI and MR breeds; in particular, GI and MR produced 0.67 and 0.60 kg/day less milk than SA, respectively (*p* < 0.05). Milk of GA had greater protein percentage than milk of GI and MA (+0.50%; *p* < 0.05). The greatest F/P was calculated for GI (1.37) and the lowest for GA (1.15) and SA (1.14). In terms of lactose percentage, GA and SA exhibited lower values than JO, MA, and MR (*p* < 0.05). Finally, SA had greater SCS (6.79 units) than GI, MA, and MR (*p* < 0.05; Table 2).

The LSM of individual, groups, and indices of milk FA profile for the different breeds are reported in Table 3. Breed affected C4:0, C14:0, C15:0, C15:0 iso and anteiso forms, C16:0, C16:1, C17:0, C17:0 iso and anteiso forms, C18:0, DI C16:0, and AI (*p* < 0.05) and the main differences were detected between SA and local breeds. Specifically, milk of SA had 0.28 g/100 g FAs more C4:0 than milk of GA; 1.56 and 3.37 g/100 g FAs more C14:0 and C16:0, respectively, and 0.50 greater AI than milk of GI; and 3.71 g/100 g FAs more C16:0 than milk of JO (*p* < 0.05). On the other hand, milk of SA had 0.28, 0.30, and 3.75 g/100 g FAs less C15:0, C17:0, and C18:0 than milk of GI, respectively; 0.23 g/100 g FAs less C17:0 than milk of JO; and 0.27, 0.28, and 2.44 g/100 g FAs less C15:0, C17:0, and C18:0 than milk of MA, respectively (*p* < 0.05). In addition, SA milk showed lower contents of iso and anteiso forms for C15:0 and C17:0 than local breeds. In detail, milk of SA had less contents of C15:0 and C17:0 iso forms than local breeds (-0.11 and -0.09 g/100 g of FAs, respectively; *p* < 0.01). Whereas, according to C15:0 and C17:0 anteiso forms SA milk produced 0.12 and 0.51 g/100 g of FAs than JO and GI, JO and Ma breed, respectively (*p* < 0.05). Among local breeds, very few differences were detected in FA profile; MR produced 0.30 and 0.27 g/100 g FAs less C16:1 than milk of GA and GI, respectively (*p* < 0.05), and 1.27, 1.36, and 1.11 lower DI C16:0 than milk of GA, GI, and JO, respectively (*p* < 0.05; Table 3).

### 3.2. Effect of Stage of Lactation

**Figure 1:**
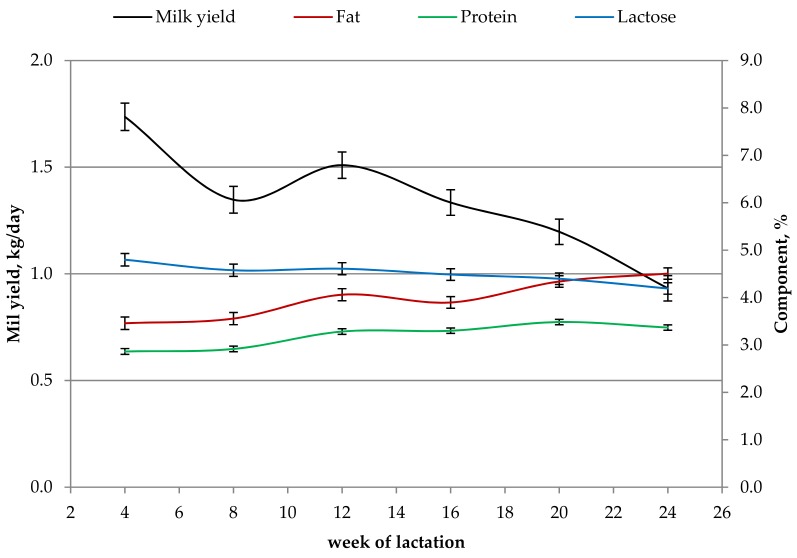
Least squares means (with standard error) of goat milk yield (black line), and fat (red line), protein (green line), and lactose (blue line) percentages throughout lactation.

Variations of milk yield, and fat, protein, and lactose percentages throughout lactation are depicted in Figure 1. The greatest daily milk production was obtained in the 4th week of lactation (peak of lactation) followed by a decrease of 0.39 kg (22%) until the 8th week of lactation (*p* < 0.001). An overall milk yield reduction of 0.80 kg (46%) was observed between the first and the last sampling week (*p* < 0.001). Protein and fat percentages showed a similar pattern throughout lactation. The lowest values of fat (3.46%) and protein (2.86%) were observed at the peak of lactation, whereas the greatest values were detected at the end of lactation (4.53% and 3.34%, respectively; *p* < 0.001). Regarding lactose percentage, the greatest (4.83%) and lowest (4.22%) values were obtained at the peak and the end of lactation, respectively. Fat to protein ratio ranged from 1.18 (4th week of lactation) to 1.34 (24th week of lactation; *p* < 0.05) and SCS was quite stable between the 4th (5.44 units) and 20th (5.37 units) week of lactation and increased to 6.34 units at the end of lactation (data not shown).

Week of lactation affected all individual, groups, and indices of FA (*p* < 0.001; Table 4). However, several fluctuations were observed throughout lactation. In particular, the greatest values of C8:0, C10:0, C12:0, C14:0, SFA, SCFA, MCFA, SFA/UFA, AI, EI, and TI were observed in the 4th, 8th, and 20th week of lactation, which differed significantly from the lowest contents in the 16th and 24th week of lactation (*p* < 0.001). In particular, the difference between the maximum and the minimum value ranged from 23% to 27% for C8:0, C14:0, SCFA, EI, and TI, from 33% to 36% for C10:0, C12:0, and SFA/UFA, and it was 8%, 15%, and 44% for SFA, MCFA, and AI, respectively. Moreover, C14:0 and MCFA contents in the 4th, 8th, and 20th week of lactation differed significantly from the content in the 12th week of lactation (Table 4). The odd FA (C15:0 and C17:0) showed the greatest amount in the 8th week of lactation and the lowest in the 12th, 20th, and 24th week of lactation; moreover, for C15:0, also the 4th week of lactation showed the lowest content. The difference between the maximum and minimum value for C15:0 and C17:0 was 31% and 24%, respectively. A peculiar pattern was detected for the n6 to n3 ratio (n6/n3) and LCFA; specifically, n6/n3 increased by 22% from 4th to 8th week of lactation, decreased by 37% from 8th to 12th week of lactation, increased by 25% from 12th to 16th week of lactation, and remained quite stable thereafter. The LCFA increased by 20% between 4th and 16th week of lactation, decreased by 16% between 16th and 20th week of lactation and increased by 15% between 20th and 24th week of lactation (*p* < 0.001).

Regarding C18:1, n3, n6, CLA, UFA, MUFA, PUFA, and DI C16:0, they were generally lower in early than late lactation (*p* < 0.001). In particular, C18:1, UFA, and MUFA contents were quite stable from 4th to 12th week of lactation, they increased by 14%, 10%, and 14% from 12th to 16th week of lactation, respectively (*p* < 0.001), decreased by similar percentages from 16th to 20th week of lactation, and peaked in the 24th week of lactation. The CLA content increased by 17% between the 12th and 16th week of lactation and peaked in the 24th week of lactation. The PUFA and n6 changed moderately between 4th and 16th week of lactation, and they increased by 20% and 21% (*p* < 0.001) from 16th to 24th week of lactation, respectively. The lowest and greatest n3 content was in the 8th and 12th week of lactation, respectively, with a difference of 57% (*p* < 0.001). The DI C18:0 was lower in the 12th and 16th compared with other sampling weeks (*p* < 0.001), and the greatest values were in the 20th and 24th weeks of lactation. The C18:2 fluctuated through the whole lactation, with the lowest and greatest content in the 16th and 24th week of lactation, respectively.

Different patterns to the previous ones were observed for C4:0, C6:0, C16:0, and C18:0. Specifically, C4:0 and C6:0 showed the greatest contents in the 12th week of lactation; nevertheless, they did not differ significantly from the contents in the 20th and 24th week of lactation in the case of C4:0, and from the content in the 4th week of lactation for C6:0. The lowest C4:0 content was obtained in the 8th week of lactation, even if it did not differ from the content in the 4th and 16th week of lactation, and the lowest C6:0 value was observed in the 24th week of lactation, but it did not differ from the content in the 8th, 16th, and 20th week of lactation. The C16:0 decreased from 4th to 16th week of lactation, increased from 16th to 20th week of lactation and decreased again thereafter. Regarding C18:0, this FA exhibited an opposite pattern to that of C16:0 throughout the lactation; in particular, its content increased from 4th to 20th week of lactation, decreased from 16th to 20th week of lactation, and increased again thereafter.

## 4. Discussion

### 4.1. Breed Effect

Only few studies have investigated the effect of goat breed on milk FA composition. Moreover, those studies have often dealt with milk fat composition of cosmopolitan breeds, likely because of their major economic interest related to greater milk yield compared with local breeds. Therefore, information on milk composition of native goat breeds is scarce, especially with regard to FA profile. However, many studies found that impact of breed effect on milk FA is lower than of the one of the diet [18,40]. Therefore, breed effect may be considered as the outcome of the adaptation of species to the environment condition (climate, feeding, and water resource) that affect milk yield and composition [41].

In the present study, as expected, the greatest milk yield was observed in the cosmopolitan breed, who is the most specialized cosmopolitan dairy breed; however, milk yield of GA, JO, and MA was similar to the one of SA breeds. Moreover, similitudes among those breeds was maintained also when milk production is standardized at 3.5% in title of fat. Fat, protein, and lactose content are in line with those reported by FAO [42], in goat milk. Fat content among breeds did not differ significantly; however, it is worth mentioning that milk of local breeds had greater fat content than milk of the cosmopolitan breed. Among local breeds, GA milk was the richest one in protein content and differed only to GI and MA breed, whereas SA breed was similar to all local breeds. Those results partially agreed with Tripaldi et al. [43] who reported lower protein content in SA milk than other local breeds and no difference among GA, MA, and MR breeds. The discrepancies to Tripaldi et al. [43] are probably due to different management conditions as local and SA breed were reared. The greater lactose content was similar among JO, MA, and MR milk breeds, whereas the lowest one was observed in GA and in SA breed. Furthermore, breeds with greatest lactose content showed lowest SCS in agreement to Sung et al. [44]. Generally, a low lactose content is usually linked to mastitis occurrence [45]. However, in the present study, the most productive goat breeds (GA, JO, MA, and SA) showed the greatest SCS. However, SCS in goat milk cannot be considered as an indicator of mastitis as in cow milk [46], due to different physiological milk secretion of the two species. In fact, milk secretion is of merocrine type in cow and apocrine type in goat [47]. The high somatic cells in goat milk is due to the great amount of cytoplasmic particles or epithelial cells passed to milk through the apocrine secretion process [47]. Fat and protein percentages of SA milk were similar to those of local breeds; this agreed with results of a study that compared SA and Portuguese local goat breeds [48]. The F/P for MR was similar to the one reported by Pizzillo et al. [30] for the same breed (1.23). However, those authors [30] reported a lower F/P for GI (1.13) and MA (1.12) breeds. These discrepancies are related to the different fat and protein content in milk considered in Pizzillo et al. [30] study.

Overall, differences of FA composition were observed between SA and the local breeds. Milk from SA showed greater C4:0, C14:0, C16:0, and AI, and lower C15:0, C15:0 iso and anteiso forms, C17:0, C17:0 iso and anteiso forms, and C18:0 contents than the local breeds. However, the greatest differences among breeds were detected for C18:0, C14:0, and C16:0. Saanen milk was poorer in C15:0, C17:0, and C18:0 and richer in C14:0 and C16:0 compared with GI and MA milk. The odd FA (C15:0 and C17:0) are considered as biomarkers of rumen activity, being ruminal bacteria population affected by diet. Stress stimuli of rumen due to the prevalence of concentrate in the diet could be the cause of an increment of anteiso and linears form of odd FA content in milk [49]. Thus, the lower levels of linears and anteiso forms of C15:0 and C17:0 in SA than local breeds suggest that odd FA contents were affected by breed as reported in Hanus et al. [50] and Bainbridge et al. [51] studies on cow breeds. The minor content of odd FA in SA milk might be related to the low adaptability of the cosmopolitan breed to the environmental condition.

The lower content of C18:0 in SA might be due to the greater desaturation activity of C18:0 (*p* = 0.07) in SA mammary gland as the result of the maintenance of balanced condition between saturated and unsaturated forms for milk fluidity control [17]. The greater AI of SA than GI suggested that milk from the local breed has lower atherogenic impact on human health [10]. In addition, a greater amount of odd FA detected in local breeds than in SA suggested that the consumption of milk from native breeds might help reduce the risk of multiple sclerosis by improving the flexibility of the plasmatic membrane [52]. Yurchenko et al. [21] indicated that the FA profile differed between SA and Swedish Landrace goat breeds for C4:0 to C16:0, C16:1, C18:1, DI C16:0, and AI. The differences between SA and GA for C4:0 and AI in the present study (0.28 g/100 g FAs and 0.24, respectively) were similar to those between SA and Swedish Landrace breeds in the study of Yurchenko et al. [21] (0.24 g/100 g FAs and 0.30, respectively). On the contrary, we reported greater C16:0 content in milk of SA than in milk of GI and JO local breeds, whereas Yurchenko et al. [21] reported lower C16:0 content in the cosmopolitan than in the Swedish Landrace native goat breeds. Finally, n6, n6/n3, and DI C18:0 were not affected by breed in Yurchenko et al. [21], which is in agreement with our study.

Among local breeds, very few differences were detected in the FA profile. For example, milk from MR had less C16:1 than milk of GA and GI. Moreover, MR had lower DI C16:0 compared with GI, GA, and JO; this suggests that mammary gland ∆-9 desaturase activity of MR was weaker than that of GA and GI. Pizzillo et al. [30] found significant differences among ricotta cheese from GI, MA, and MR in terms of C10:0, C15:0, C16:0, C18:2, SFA, and PUFA. In particular, ricotta cheese from GI milk had greater concentration of C15:0, C18:2, and PUFA, but poorer C10:0 content than milk of MA and C16:0 content than MR and MA milk [30]. Talpur et al. [53] studied the effect of Kamori and Pateri breeds (Pakistan local goat breeds) reared in the same farm and under the same feeding conditions on milk FA profile; those authors observed differences between those two breeds in terms of C6:0 to C18:0, C16:1, C18:1, SFA, MUFA, and CLA, whereas they did not report differences in terms of C4:0, C17:0, and PUFA.

### 4.2. Effect of Stage of Lactation

The pattern of milk yield and gross composition through lactation are in agreement with several studies on goat milk [20,27]. Milk yield decreased through lactation due to the reduction of efficiency in the mammary gland synthesis [54] and the deterioration of the quality of the pasture during summer season [55]. The lowest concentration of milk protein and fat in the 4th week of lactation, and the greatest at the end of lactation have been already associated to a dilution and a concentration effect, respectively [56]. Moreover, Kawas et al. [57] demonstrated that a greater forage to concentrate ratio in the ration affects milk fat, i.e., greater milk fat was observed when the ration included 75% instead of 45% of forage. The average F/P and its range were in agreement with other studies on goat milk [30,58]. The average F/P, which is an indicator of the metabolic status of the animal, was quite constant during lactation, suggesting the absence of metabolic disorders during the study [59].

The effect of week of lactation on goat milk FA composition has been reported by several authors; however, the pattern described for FA across lactation is controversial [60]. In the present study, week of lactation highly affected all individual, groups, and indices of FA, similarly to Kuchtik et al. [61], who studied the FA profile in milk of Brown Short-haired goat breed from 62 to 258 days in milk (from April to October). On the other hand, StrzaŁkowska et al. [20] did not observe an effect of stage of lactation on C4:0, C14:0, C16:0, C17:0, and MCFA. The greatest content of C8:0 to C16:0, and consequently of total SFA, in early than late lactation could be related to greater administration of supplementary concentrate in early lactation, which is responsible for an increase of de novo FA synthesis [18]. The greater SCFA content in early lactation suggested a positive energy balance, whereas the low amount of SCFA in late lactation was probably due to the greater content of LCFA, which have an important inhibitory effect on the synthesis of SCFA [13]. The greater content of LCFA in late lactation could be a consequence of (i) lower amount of concentrates administered compared with early lactation, (ii) mobilization of LCFA from adipose tissues (mainly C16:0 and C18:0) due to negative energy balance, or (iii) a combination of the two previous conditions [13]. Moreover, in late lactation, due to lower energy requirements for milk production, the LCFA with long carbon chain undergo an hydrogenation process in the mammary gland [62]. In fact, in the present study, the greater value of DI C16:0 and DI C18:0 in late lactation suggests that ∆-9 desaturase activity of mammary gland was higher at the end of lactation.

The C4:0 and C6:0 showed different patterns to other SCFA, which could be explained by the way they are synthesized. Milk C4:0 derives from malonyl-CoA (by condensation of acetyl units) but it is also uptaken by the blood as preformed C4:0 [63]. Milk C6:0 derives from the addition of only one unit of acetyl via malonyl-CoA, thus, it is less influenced by acetyl-CoA availability [63]. The odd FA amount oscillated through weeks of lactation, which is probably related to the amount of bacteria that leave the rumen being affect by the feeding ratio. A correct feeding regime based on animal requirements promotes the bacterial (containing odd FA in the lipid membrane) numerical growth in the rumen. In fact, a consequence of excessive bacterial growth causes a greater bacteria escaping from the rumen which increases the content of those FA in milk [60]. The greater content of n3, n6, CLA, MUFA, and PUFA in late lactation could be due to the greater intake of pasture than in early lactation where the forage to concentrate ratio was lower. Although n6/n3 fluctuated through lactation, it was usually maintained in the recommended ratio (4:1) for the prevention of cardiovascular diseases [64]. The greatest SFA/UFA in early lactation and in the 12th and 20th week of lactation is supported by the lower activity of desaturation of mammary gland detected in the same weeks of lactation. The AI and TI are considered as nutritional indices of lipids and their estimation allows to evaluate the effect of each food on the risk of developing coronary heart disease; in detail, the AI refers to the FA aggregation forming a plaque in the arteries, whereas TI refers to the tendency to form clots in the blood vessels [65]. In general, milk richer in UFA shows a lower AI and TI suggesting that milk could be less harmful to human health. In this study, the greater forage intake in mid and late than early lactation reduced the AI and TI improving the milk FA quality [66].

Some discrepancies were detected comparing results of the present study with findings of previous research [20,61]. In detail, discrepancies to Kuchtík et al. [61] could be related to the different feeding strategy used in their study; indeed, feed ratio included besides pasture, meadow hay, and mineral lick ad libitum, an administration of organic rolled oats and organic feed mixture (0.5 kg/day per doe, respectively). On the other hand, similitudes to StrzaŁkowska et al. [20] could be related to the feeding strategies formulated according to animal requirements similarly to the present research. However in the StrzaŁkowska et al. [20] study, besides the concentrate, from October to May [20], the feeding ratio was integrated with corn silage, hay, carrot, whereas from June to September hay was substituted with fresh grass. In detail, the C4:0 pattern in our study differed to that reported by Kuchtík et al. [61], who detected the greatest and lowest amount in early and late lactation, respectively. However, the amounts of C4:0 in our study were greater than those observed by Kuchtík et al. (1.43 g/100 g FAs) [61] and StrzaŁkowska et al. (1.27 g/100 FAs) [20] across lactation. Moreover, the pattern of C6:0 to C10:0 and CLA through lactation resembled that of Kuchtík et al. [61] and StrzaŁkowska et al. [20]. The C6:0 to C10:0 are responsible for goat flavor of dairy products [67,68] and thus the greater content of C6:0 to C10:0 in milk in the first than the second half of lactation might be responsible for stronger goaty flavor in dairy products derived from milk collected in early lactation [69]. In addition, the trend for C16:0 and C16:1 in the present study was different than that reported by Kuchtík et al. [61]. In particular, Kuchtík et al. [61] found a stable content until the 13th week of lactation, followed by an increment in the 18th week of lactation and a decrease in the 23rd week of lactation. However, considering the whole lactation period (62 to 258 days in milk), Kuchtík et al. [61] observed the greatest content of C16:0 and C16:1 in late lactation, as reported in our study; this similarity could suggesting the effect of stage of herbage maturity on milk FA. On the other hand, the pattern of C16:1 observed in our study was similar to that of StrzaŁkowska et al. [20], who found a lower amount in early than late lactation probably due to the inclusion of fresh grass from June to September. However, Kuchtík et al. [61] found a different trend than that reported in the current study for C12:0 and C14:0; those authors detected the lowest values between 9th and 13th and in the 23rd week of lactation, and the greatest content in the 18th week of lactation. Considering the odd FA, C15:0 pattern differed to that of Kuchtík et al. [61], who found a lower content in the 9th week of lactation and greater content between 13th and 18th week of lactation, and reporting the lowest content in the 23rd week of lactation. The C18:0 and C18:1 patterns differed to those of Kuchtík et al. [61], who observed greater amount in the 9th, 13th, and 23rd week of lactation, and lowest content in the 18th week of lactation. On the contrary, StrzaŁkowska et al. [20] reported greatest values of C18:0 and C18:1 in early and mid than late lactation. Those discrepancies may depend by the different feeding strategy adopted in each study. The trend of C18:2 was similar to the one reported by StrzaŁkowska et al. [20], who found lower content in early than mid and late lactation. An opposite situation was described by Kuchtík et al. [61], who showed the greatest content in the 9th week of lactation and the lowest at the end of lactation. The SCFA trend was similar to Strzałkowska et al. [20], however, they reported a greater amount of SCFA in early (29.22 g/100 g FAs) and in late (24.38 g/100 g FAs) lactation compared with the present study. Strzałkowska et al. [20] reported a similar trend for LCFA but with lower content than the present study (30.18 to 33.60 g/100 g FAs). The MCFA showed the greatest content in early lactation and in the 20th week of lactation, contrary to Strzałkowska et al. [20], who reported similar amount across lactation.

## 5. Conclusions

Results of the present study showed that goat milk FA profile was affected by breed and stage of lactation. Breeds differed for C4:0, C14:0, C15:0, C15:0 iso and anteiso forms, C16:0, C16:1, C17:0, C17:0 iso and anteiso forms, C18:0, DI C16:0, and AI. The main differences of FA composition were detected between SA and some local breeds. Saanen milk was richer in C4:0, C14:0, and C16:0 than milk of GA, GI, and GI and JO, respectively. In addition, the greater AI in SA milk than GI milk breed and the lower contents of odd FA in SA milk than local breeds, may suggest less adaptability of the cosmopolitan breed to the environmental condition than of its origins. Thus, milk of local breeds may have a greater potential benefit on human health. Very few differences were detected within local breeds. Week of lactation affected significantly all individual, groups, and indices of FA. However, the pasture composition and stage of herbage maturity could affect FA content showing peculiar patterns through lactation. In particular, individual SCFA and some MCFA were more abundant in early than late lactation probably due to the greater amount of concentrate administered in the onset of lactation than in later stages. The greatest desaturation activity of mammary gland was observed at the end of lactation, as suggested by DI C16:0 and DI C18:0. Contrary to SFA, the amount of n3, n6, CLA, UFA, MUFA, and PUFA were greater at the end than at the beginning of lactation. These differences are linked to the increase of forage intake in mid and toward the end of lactation. In fact, late lactation is characterized by low concentrate administration in favor of the pasture. In conclusion, this study offers a contribute to the characterization and comparison of FA profile of milk from local breeds and a cosmopolitan breed under the same farming conditions, evidencing that milk from local breeds could be valorized for its better FA profile may have a greater potential benefit on human health compared to milk of the cosmopolitan breed.

## Figures and Tables

**Table 1:** Chemical composition and energy content of the hay and the concentrate administered to the goats during the trial.

Trait ^1^	Hay	Commercial Concentrate
DM (%)	89.1	88.2
CP (% of DM)	15.1	21.7
Fat (% of DM)	1.9	3.5
NSC (% of DM)	20.7	42.7
Cellulose (% of DM)	29.5	10.5
Ash (% of DM)	9.5	9.1
NDF (% of DM)	52.6	23.0
ADF (% of DM)	36.6	9.0
ADL (% of DM)	3.9	3.3
NEL (Mcal/kg DM)	1.10	1.77

^1^ DM, dry matter; CP, crude protein; NSC, nonstructural carbohydrates; NDF, neutral detergent fibre; ADF, acid detergent fibre; ADL, acid detergent lignin; NEL, net energy of lactation.

**Table 2:** Least squares means of milk yield and composition of 6 goat breeds ^1^.

Traits	Breed						Overall		
	GA	GI	JO	MA	MR	SA	Mean	SEM	*p*
Milk yield (kg/day)	1.26 ^ab^	1.06 ^b^	1.48 ^ab^	1.39 ^ab^	1.13 ^b^	1.73 ^a^	1.34	0.11	<0.001
FCM3.5% (kg/day)	1.32 ^ab^	1.14 ^b^	1.45 ^ab^	1.43 ^ab^	1.16 ^b^	1.67 ^a^	1.36	0.10	<0.001
Fat (%)	4.15	4.20	4.01	3.92	3.92	3.61	3.97	0.18	0.310
Protein (%)	3.52 ^a^	3.02 ^b^	3.20 ^ab^	3.02 ^b^	3.27 ^ab^	3.20 ^ab^	3.20	0.10	0.012
Lactose (%)	4.31 ^b^	4.55 ^ab^	4.58 ^a^	4.59 ^a^	4.69 ^a^	4.34 ^b^	4.51	0.05	<0.001
F/P	1.15 ^b^	1.37 ^a^	1.25 ^ab^	1.30 ^ab^	1.21 ^ab^	1.14 ^b^	1.22	0.03	0.005
SCS (units)	5.77 ^a^	4.20 ^b^	5.34 ^ab^	4.93 ^b^	5.10 ^b^	6.79 ^a^	5.35	0.34	<0.001

Abbreviations: GA, Garganica; GI, Girgentana; JO, Jonica; MA, Maltese; MR, Mediterranean Red; SA, Saanen; FCM 3.5%, fat-corrected milk at 3.5%; F/P, fat to protein ratio; SCS, somatic cell score; SEM, standard error of the mean. ^1^ Least squares means with different superscript letters within a row are significantly different (*p* < 0.05).

**Table 3:** Least squares means of individual, groups, and indices of milk fatty acid profile (g/100 g of total identified fatty acids) of 6 goat breeds ^1^.

Fatty Acid	Breed						Overall		
	GA	GI	JO	MA	MR	SA	Mean	SEM	*P*
C4:0	1.89 ^b^	2.03 ^ab^	2.01 ^ab^	2.00 ^ab^	2.09 ^ab^	2.17 ^a^	2.03	0.06	**0.043**
C6:0	2.19	2.22	2.35	2.24	2.37	2.26	2.27	0.07	0.330
C8:0	2.65	2.58	2.90	2.66	2.79	2.58	2.69	0.10	0.189
C10:0	8.44	7.84	9.01	8.24	8.73	8.22	8.41	0.34	0.186
C12:0	3.43	3.14	3.85	3.44	3.46	3.31	3.44	0.16	0.065
C14:0	8.57 ^ab^	7.69 ^b^	8.47 ^ab^	8.50 ^ab^	8.67 ^ab^	9.25 ^a^	8.52	0.30	**0.029**
C15:0	1.72 ^ab^	1.86 ^a^	1.82 ^ab^	1.85 ^a^	1.73 ^ab^	1.58 ^b^	1.76	0.05	**0.013**
C15:0 iso	0.42 ^a^	0.40 ^a^	0.41 ^a^	0.43 ^a^	0.42 ^a^	0.31 ^b^	0.40	0.02	**0.009**
C15:0 anteiso	0.35 ^ab^	0.35 ^ab^	0.40 ^a^	0.37 ^ab^	0.35 ^ab^	0.28 ^b^	0.35	0.02	**0.018**
C16:0	23.74 ^ab^	22.48 ^b^	22.14 ^b^	23.46 ^ab^	24.19 ^ab^	25.85 ^a^	23.54	0.71	**0.014**
C16:1	1.01 ^a^	0.98 ^a^	0.90 ^ab^	0.86 ^ab^	0.71 ^b^	0.87 ^ab^	0.89	0.05	**0.002**
C17:0	1.87 ^ab^	2.02 ^a^	1.95 ^a^	2.00 ^a^	1.89 ^ab^	1.72 ^b^	1.91	0.04	**0.002**
C17:0 iso	0.63 ^a^	0.68 ^a^	0.67 ^a^	0.66 ^a^	0.63 ^a^	0.56 ^b^	0.64	0.01	**<0.001**
C17:0 anteiso	0.49 ^ab^	0.52 ^a^	0.50 ^a^	0.51 ^a^	0.48 ^ab^	0.43 ^b^	0.49	0.01	**0.003**
C18:0	13.85 ^ab^	15.60 ^a^	14.16 ^ab^	14.29 ^a^	13.65 ^ab^	11.85 ^b^	13.90	0.54	**0.002**
C18:1	23.32	24.59	22.95	23.06	22.68	22.78	23.23	0.69	0.359
C18:1trans-11	1.84	2.09	2.03	2.00	1.92	2.03	1.98	0.10	0.593
CLAc9 trans-11	0.73	0.73	0.73	0.73	0.72	0.85	0.75	0.03	0.119
C18:2	3.00	3.17	3.20	3.18	2.87	3.31	3.12	0.10	0.083
n3	0.86	1.00	0.96	0.99	0.86	0.92	0.93	0.04	0.069
n6	4.30	4.36	4.37	4.41	4.07	4.72	4.37	0.13	0.063
CLA	0.91	0.88	0.88	0.91	0.88	1.05	0.92	0.04	0.068
SFA	70.23	69.14	70.46	71.00	70.91	70.35	70.35	0.65	0.346
UFA	30.16	31.59	29.86	29.97	28.97	29.93	30.08	0.77	0.280
MUFA	24.84	25.53	24.21	23.56	24.11	24.03	24.38	0.55	0.147
PUFA	5.15	5.36	5.33	5.40	4.97	5.65	5.31	0.15	0.102
SCFA	15.28	14.79	16.39	15.25	16.12	15.34	15.53	0.50	0.194
MCFA	38.96	36.34	37.67	38.52	38.87	40.34	38.45	0.87	0.059
LCFA	45.77	48.64	45.84	46.13	44.64	43.36	45.73	1.14	0.057
n6/n3	5.20	4.56	4.67	4.63	4.93	5.36	4.89	0.19	0.070
SFA/UFA	2.36	2.21	2.39	2.40	2.51	2.43	2.38	0.08	0.207
DI C16:0	4.09 ^a^	4.18 ^a^	3.93 ^a^	3.51 ^ab^	2.82 ^b^	3.32 ^ab^	3.64	0.22	**<0.001**
DI C18:0	62.93	61.41	62.10	61.83	62.51	65.73	62.75	1.00	0.073
AI	2.08 ^ab^	1.82 ^b^	2.05 ^ab^	2.10 ^ab^	2.22 ^ab^	2.32 ^a^	2.10	0.10	**0.035**
EI	5.63	5.00	5.59	5.37	5.29	5.30	5.36	0.22	0.342
TI	2.71	2.55	2.62	2.73	2.80	2.80	2.71	0.09	0.258

Abbreviations: GA, Garganica; GI, Girgentana; JO, Jonica; MA, Maltese; MR, Mediterranean Red; SA, Saanen; CLA, conjugated linoleic acids; SFA, saturated fatty acids; UFA, unsaturated fatty acids; MUFA, monounsaturated fatty acids; PUFA, polyunsaturated fatty acids; SCFA, short chain fatty acids; MCFA, medium chain fatty acids; LCFA, long chain fatty acids; DI C16:0, desaturation index of C16:0; DI C18:0, desaturation index of C18:0; AI, atherogenicity index; EI, elongation index; TI, thrombogenic index; SEM, standard error of the mean. ^1^ Least squares means with different superscript letters within a row are significantly different (*p* < 0.05). In bold, *p*-values lower than 0.05.

**Table 4:** Least squares means of individual, groups (g/100 g of total identified fatty acids), and indices of goat milk fatty acids throughout lactation ^1^ (*p* < 0.001).

Fatty Acid	Week of Lactation						Overall	
	4	8	12	16	20	24	Mean	SEM
C4:0	1.96 ^bc^	1.92 ^c^	2.16 ^a^	2.01 ^bc^	2.09 ^ab^	2.05 ^ab^	2.03	0.04
C6:0	2.38 ^ab^	2.24 ^bc^	2.44 ^a^	2.14 ^c^	2.33 ^bc^	2.09 ^c^	2.27	0.05
C8:0	2.93 ^a^	2.73 ^ab^	2.92 ^a^	2.48 ^c^	2.79 ^ab^	2.31 ^c^	2.69	0.07
C10:0	9.47 ^a^	8.89 ^a^	8.81 ^a^	7.37 ^b^	8.96 ^a^	6.98 ^b^	8.41	0.26
C12:0	3.75 ^a^	3.73 ^a^	3.46 ^ab^	3.11 ^bc^	3.76 ^a^	2.83 ^c^	3.44	0.12
C14:0	9.51 ^a^	9.31 ^a^	8.11 ^b^	7.52 ^c^	8.81 ^a^	7.88 ^bc^	8.52	0.19
C15:0	1.69 ^c^	2.10 ^a^	1.60 ^c^	1.90 ^b^	1.61 ^c^	1.64 ^c^	1.76	0.04
C15:0 iso	0.37 ^b^	0.50 ^a^	0.38 ^b^	0.45 ^a^	0.33 ^b^	0.36 ^b^	0.40	0.02
C15:0 anteiso	0.32 ^b^	0.44 ^a^	0.33 ^b^	0.40 ^a^	0.28 ^b^	0.33 ^b^	0.35	0.01
C16:0	24.63 ^ab^	23.75 ^b^	22.48 ^c^	21.52 ^c^	25.61 ^a^	23.88 ^b^	23.60	0.44
C16:1	0.82 ^b^	0.90 ^ab^	0.77 ^b^	0.88 ^ab^	0.92 ^ab^	1.04 ^a^	0.89	0.04
C17:0	1.95 ^bc^	2.14 ^a^	1.80 ^cd^	2.00 ^ab^	1.72 ^d^	1.84 ^cd^	1.91	0.04
C17:0 iso	0.59 ^b^	0.67 ^a^	0.63 ^b^	0.67 ^a^	0.62 ^b^	0.64 ^b^	0.64	0.01
C17:0 anteiso	0.51 ^b^	0.59 ^a^	0.46 ^bc^	0.52 ^b^	0.41 ^c^	0.44 ^c^	0.49	0.01
C18:0	12.43 ^cd^	13.05 ^cd^	15.75 ^b^	17.13 ^a^	11.75 ^d^	13.28 ^c^	13.90	0.35
C18:1	21.61 ^c^	21.98 ^c^	22.38 ^b^	25.50 ^a^	22.00 ^b^	25.91 ^a^	23.23	0.52
C18:1trans-11	1.75 ^c^	1.61 ^c^	2.03 ^b^	2.28 ^a^	1.94 ^b^	2.29 ^a^	1.98	0.07
CLAc9 trans-11	0.57 ^c^	0.52 ^c^	0.67 ^c^	0.83 ^b^	0.86 ^ab^	1.06 ^a^	0.75	0.03
C18:2	2.94 ^bc^	3.20 ^ab^	3.04 ^bc^	2.88 ^c^	3.21 ^ab^	3.46 ^a^	3.12	0.08
n3	0.82 ^b^	0.70 ^c^	1.10 ^a^	0.88 ^b^	1.06 ^a^	1.04 ^a^	0.93	0.03
n6	4.02 ^c^	4.25 ^bc^	4.16 ^c^	4.19 ^c^	4.55 ^b^	5.06 ^a^	4.37	0.09
CLA	0.74 ^cd^	0.68 ^d^	0.83 ^c^	1.00 ^b^	1.03 ^b^	1.23 ^a^	0.92	0.03
SFA	72.79 ^a^	71.64 ^a^	71.30 ^a^	68.17 ^b^	71.01 ^a^	67.17 ^b^	70.35	0.49
UFA	27.95 ^b^	28.55 ^b^	29.03 ^b^	32.02 ^a^	29.19 ^b^	33.74 ^a^	30.08	0.60
MUFA	22.43 ^b^	23.41 ^b^	23.43 ^b^	26.67 ^a^	23.38 ^b^	26.97 ^a^	24.38	0.43
PUFA	4.86 ^c^	4.96 ^c^	5.26 ^bc^	5.07 ^c^	5.61 ^ab^	6.10 ^a^	5.31	0.11
SCFA	16.86 ^a^	15.90 ^a^	16.44 ^a^	14.13 ^b^	16.33 ^a^	13.52 ^b^	15.53	0.37
MCFA	40.44 ^a^	40.27 ^a^	36.91 ^b^	35.10 ^c^	40.56 ^a^	37.43 ^b^	38.45	0.54
LCFA	42.26 ^c^	43.85 ^c^	46.63 ^b^	50.51 ^a^	42.36 ^c^	48.76 ^ab^	45.73	0.82
n6/n3	5.04 ^bc^	6.16 ^a^	3.86 ^d^	4.81 ^bc^	4.49 ^c^	4.99 ^bc^	4.89	0.14
SFA/UFA	2.67 ^a^	2.54 ^a^	2.48 ^a^	2.15 ^b^	2.47 ^a^	1.99 ^b^	2.38	0.07
DI C16:0	3.27^c^	3.65 ^bc^	3.27 ^c^	3.96 ^ab^	3.49 ^bc^	4.21 ^a^	3.64	0.18
DI C18:0	63.45^bc^	62.79 ^c^	58.72 ^d^	60.06 ^d^	65.28 ^ab^	66.20 ^a^	62.75	0.65
AI	2.48^a^	2.30 ^a^	2.06 ^b^	1.72 ^c^	2.26 ^ab^	1.75 ^c^	2.10	0.07
EI	5.95^a^	5.90 ^a^	5.04 ^b^	4.90 ^b^	5.55 ^a^	4.83 ^b^	5.36	0.14
TI	3.02^a^	2.88 ^ab^	2.71 ^bc^	2.53 ^cd^	2.71 ^bc^	2.38 ^d^	2.71	0.06

Abbreviations: CLA, conjugated linoleic acids; SFA, saturated fatty acids; UFA, unsaturated fatty acids; MUFA, monounsaturated fatty acids; PUFA, polyunsaturated fatty acids; SCFA, short chain fatty acids; MCFA, medium chain fatty acids; LCFA, long chain fatty acids; DI C16:0, desaturation index of C16:0; DI C18:0, desaturation index of C18:0; AI, atherogenicity index; EI, elongation index; TI, thrombogenic index; SEM, standard error of the mean. ^1^ Least squares means with different superscript letters within a row are significantly different (*p* < 0.05).

## References

[1] FAOSTAT; The Food and Agriculture Organization Corporate Statistical Database. www.fao.org/faostat/en/.

[2] Boyazoglu J., Morand-Fehr P. (2001). Mediterranean dairy sheep and goat products and their quality A critical review. Small Rumin. Res..

[3] Lordan R., Tsoupras A., Mitra B., Zabetakis I. (2018). Dairy fats and cardiovascular disease: Do we really need to be concerned?. Foods.

[4] Jirillo F., Jirillo E., Magrone T. (2010). Donkey’s and Goat’s Milk Consumption and Benefits to Human Health with Special Reference to the Inflammatory Status. Curr. Pharm. Des..

[5] Faye B., Konuspayeva G. (2012). The sustainability challenge to the dairy sector—The growing importance of non-cattle milk production worldwide. Int. Dairy J..

[6] Park Y.W. (1994). Hypo-allergenic and therapeutic significance of goat milk. Small Rumin. Res..

[7] Manuelian C.L., Currò S., Penasa M., Cassandro M., De Marchi M. (2017). Characterization of major and trace minerals, fatty acid composition, and cholesterol content of Protected Designation of Origin cheeses. J. Dairy Sci..

[8] Calder P.C. (2015). Functional Roles of Fatty Acids and Their Effects on Human Health. J. Parenter. Enter. Nutr..

[9] Walther B., Schmid A., Sieber R., Wehrmüller K. (2008). Cheese in nutrition and health. Dairy Sci. Technol..

[10] Poppitt S.D., Koegh G.F., Mulvey T.B., McArdle B.H., MacGibbon A.K.H., Cooper G.J.S. (2002). Lipid-loweing effects of a modified butter-fat: A controlled intervention trial in healthy men. Eur. J. Clin. Nutr..

[11] Schönfeld P., Wojtczak L. (2016). Short- and medium-chain fatty acids in energy metabolism: The cellular perspective. J. Lipid Res..

[12] Van Schalkwijk D.B., Pasman W.J., Hendriks H.F.J., Verheij E.R., Rubingh C.M., Van Bochove K., Vaes W.H.J., Adiels M., Freidig A.P., De Graaf A.A. (2014). Dietary medium chain fatty acid supplementation leads to reduced VLDL lipolysis and uptake rates in comparison to linoleic acid supplementation. PLoS ONE.

[13] Chilliard Y., Ferlay A., Mansbridge R.M., Doreau M. (2000). Ruminant milk fat plasticity: Nutritional control of saturated, polyunsaturated, trans and conjugated fatty acids. Ann. Zootech..

[14] Palmquist D.L. (2009). Milk fat: Origin of fatty acids and influence of nutritional factors thereon. Adv. Dairy Chem..

[15] Benedet A., Manuelian C.L., Zidi A., Penasa M., De Marchi M. (2019). Invited review: β-hydroxybutyrate concentration in blood and milk and its associations with cow performance. Animal.

[16] Griinari J.M., Dwyer D.A., McGuire M.A., Bauman D.E., Palmquist D.L., Nurmela K.V.V. (1998). Trans-Octadecenoic Acids and Milk Fat Depression in Lactating Dairy Cows. J. Dairy Sci..

[17] Gama M.A.S., Garnsworthy P.C., Griinari J.M., Leme P.R., Rodrigues P.H.M., Souza L.W.O., Lanna D.P.D. (2008). Diet-induced milk fat depression: Association with changes in milk fatty acid composition and fluidity of milk fat. Livest. Sci..

[18] Nantapo C.T.W., Muchenje V., Hugo A. (2014). Atherogenicity index and health-related fatty acids in different stages of lactation from Friesian, Jersey and Friesian × Jersey cross cow milk under a pasture-based dairy system. Food Chem..

[19] Solaiman S.G. (2010). Goat science and production.

[20] Strzalkowska N., Jóźwik A., Bagnicka E., Krzyzewski J., Horbańczuk K., Pyzel B., Horbańczuk J.O. (2009). Chemical composition, physical traits and fatty acid profile of goat milk as related to the stage of lactation. Anim. Sci. Pap. Rep..

[21] Yurchenko S., Sats A., Tatar V., Kaart T., Mootse H., Jõudu I. (2018). Fatty acid profile of milk from Saanen and Swedish Landrace goats. Food Chem..

[22] Di Trana A., Sepe L., Di Gregorio P., Di Napoli M.A., Giorgio D., Caputo A.R., Claps S., Vastola A. (2015). The Role of Local Sheep and Goat Breeds and Their Products as a Tool for Sustainability and Safeguard of the Mediterranean Environment. The Sustainability of Agro-Food and Natural Resource Systems in the Mediterranean Basin.

[23] Malhado C.H.M., Carneiro P.L.S., Affonso P.R.A.M., Souza A.A.O., Sarmento J.L.R. (2009). Growth curves in Dorper sheep crossed with the local Brazilian breeds, Morada Nova, Rabo Largo, and Santa Inês. Small Rumin. Res..

[24] Benjelloun B., Alberto F.J., Streeter I., Boyer F., Coissac E., Stucki S., BenBati M., Ibnelbachyr M., Chentouf M., Bechchari A. (2015). Characterizing neutral genomic diversity and selection signatures in indigenous populations of Moroccan goats (Capra hircus) using WGS data. Front. Genet..

[25] DAD-IS Domestic Animal Diversity Information System (DAD-IS). http://dad.fao.org/.

[26] Gandini G.C., Villa E. (2003). Analysis of the cultural value of local livestock breeds: A methodology. J. Anim. Breed. Genet..

[27] Currò S., Manuelian C.L., De Marchi M., De Palo P., Claps S., Maggiolino A., Campanile G., Rufrano D., Fontana A., Pedota G. (2019). Autochthonous dairy goat breeds showed better milk quality than Saanen under the same environmental conditions. Arch. Anim. Breed..

[28] Tufarelli V., Dario M., Laudadio V. (2009). Forage to concentrate ratio in Jonica breed goats: Influence on lactation curve and milk composition. J. Dairy Res..

[29] Albenzio M., Caroprese M., Marino R., Muscio A., Santillo A., Sevi A. (2006). Characteristics of Garganica goat milk and Cacioricotta cheese. Small Rumin. Res..

[30] Pizzillo M., Claps S., Cifuni G.F., Fedele V., Rubino R. (2004). Effect of goat breed on the sensory, chemical and nutritional characteristics of ricotta cheese. Livest. Prod. Sci..

[31] Sacchi P., Chessa S., Budelli E., Bolla P., Ceriotti G., Soglia D., Rasero R., Cauvin E., Caroli A. (2005). Casein Haplotype Structure in Five Italian Goat Breeds. J. Dairy Sci..

[32] Currò S., De Marchi M., Claps S., Salzano A., De Palo P., Manuelian C.L., Neglia G. (2019). Differences in the Detailed Milk Mineral Composition of Italian Local and Saanen Goat Breeds. Animals.

[33] Villaquiran M., Gipson T.A., Merkel R., Goetsch A., Sahlu T. (2005). Body Condition Scoring for Improved Management. Proceeding of the 20th Annual Goat Field Day.

[34] National Research Council (2007). Nutrient Requirements of Small Ruminants: Sheep, Goats, Cervids, and New World Camelids.

[35] Pulina G., Cannas A., Serra A., Vallebella R. Determination and estimation of the energy value in Sardinian goat milk. Proceedings of the Congress of Società Italiana Scienze Veterinarie (SISVet).

[36] Wiggans G.R., Shook G.E. (1987). A Lactation Measure of Somatic Cell Count. J. Dairy Sci..

[37] Christie W.W. (1993). Preparation of Ester Derivatives of Fatty Acids for Chromatographic Analysis. Advanes Lipid Methodoly II.

[38] Čejna V., Chládek G. (2005). The importance of monitoring changes in milk fat to milk protein ratio in Holstein cows during lactation. J. Cent. Eur. Agric..

[39] Paura L., Jonkus D., Ruska D. (2012). Evaluation of the milk fat to protein ratio and fertility traits in Latvian brown and Holstein dairy cows. Acta Agric. Slov. Suppl..

[40] Roca Fernandez A.I., Gonzalez Rodriguez A. (2012). Effect of Dietary and Animal Factors on Milk Fatty Acids Composition of Grazing Dairy Cows: A Review. Iran. J. Appl. Anim. Sci..

[41] Claps S., Roberta R., Di Trana A., di Napoli M.A., Giorgio D., Sepe L. (2018). Bioactive compounds in goat milk and cheese: The role of feeding system and breed. Goat Science.

[42] Food and Agriculture Organization of the United Nations (FAO) (2013). Milk and dairy products in human nutrition. Milk and Dairy Products in Human Nutrition.

[43] Tripaldi C., Martillotti F., Terramoccia S. (1998). Content of taurine and other free amino acids in milk of goats bred in Italy. Small Rumin. Res..

[44] Sung Y.Y., Wu T.I., Wang P.H. (1999). Evaluation of milk quality of Alpine, Nubian, Saanen and Toggenburg breeds in Taiwan. Small Rumin. Res..

[45] Raynal-Ljutovac K., Pirisi A., de Crémoux R., Gonzalo C. (2007). Somatic cells of goat and sheep milk: Analytical, sanitary, productive and technological aspects. Small Rumin. Res..

[46] Barrón-Bravo O.G., Gutiérrez-Chávez A.J., Ángel-Sahagún C.A., Montaldo H.H., Shepard L., Valencia-Posadas M. (2013). Losses in milk yield, fat and protein contents according to different levels of somatic cell count in dairy goats. Small Rumin. Res..

[47] Jiménez-Granado R., Sánchez-Rodríguez M., Arce C., Rodríguez-Estévez V. (2014). Factors affecting somatic cell count in dairy goats: A review. Spanish J. Agric. Res..

[48] Trancoso I.M., Trancoso M.A., Martins A.P.L., Roseiro L.B. (2010). Chemical composition and mineral content of goat milk from four indigenous Portuguese breeds in relation to one foreign breed. Int. J. Dairy Technol..

[49] Vlaeminck B., Fievez V., Cabrita A.R.J., Fonseca A.J.M., Dewhurst R.J. (2006). Factors affecting odd- and branched-chain fatty acids in milk: A review. Anim. Feed Sci. Technol..

[50] Hanus O., Samkova E., Křížova L., Hasoňova L., Kala R. (2018). Role of fatty acids in milk fat and the influence of selected factors on their variability—a review. Molecules.

[51] Bainbridge M.L., Cersosimo L.M., Wright A.D.G., Kraft J. (2016). Rumen bacterial communities shift across a lactation in Holstein, Jersey and Holstein × Jersey dairy cows and correlate to rumen function, bacterial fatty acid composition and production parameters. FEMS Microbiol. Ecol..

[52] Jenkins B., West J.A., Koulman A. (2015). A review of odd-chain fatty acid metabolism and the role of pentadecanoic acid (C15:0) and heptadecanoic acid (C17:0) in health and disease. Molecules.

[53] Talpur F.N., Bhanger M.I., Memon N.N. (2009). Milk fatty acid composition of indigenous goat and ewe breeds from Sindh, Pakistan. J. Food Compos. Anal..

[54] Albenzio M., Santillo A., Caroprese M., Ciliberti M.G., Marino R., Sevi A. (2016). Effect of stage of lactation on the immune competence of goat mammary gland. J. Dairy Sci.

[55] Sitzia M., Bonanno A., Todaro M., Cannas A., Atzori A.S., Francesconi A.H.D., Trabalza-Marinucci M. (2015). Feeding and management techniques to favour summer sheep milk and cheese production in the Mediterranean environment. Small Rumin. Res..

[56] Goetsch A.L., Zeng S.S., Gipson T.A. (2011). Factors affecting goat milk production and quality. Small Rumin. Res..

[57] Kawas J.R., Lopes J., Danelon D.L., Lu C.D. (1991). Influence of forage-to-concentrate ratios on intake, digestibility, chewing and milk production of dairy goats. Small Rumin. Res..

[58] Desnoyers M., Duvaux-Ponter C., Rigalma K., Roussel S., Martin O., Giger-Reverdin S. (2008). Effect of concentrate percentage on ruminal pH and time-budget in dairy goats. Animal.

[59] Vlček M., Žitný J., Kasarda R. (2016). Changes of fat-to-protein ratio from start to the mid- lactation and the impact on milk yield. J. Cent. Eur. Agric..

[60] Craninx M., Steen A., Van Laar H., Van Nespen T., Martín-Tereso J., De Baets B., Fievez V. (2008). Effect of Lactation Stage on the Odd- and Branched-Chain Milk Fatty Acids of Dairy Cattle Under Grazing and Indoor Conditions. J. Dairy Sci..

[61] Kuchtík J., KrálÍčková Š., Zapletal D., Węglarzy K., Šustová K., Skrzyżala I. (2015). Changes in physico-chemical characteristics, somatic cell count and fatty acid profile of brown short-haired goat milk during lactation. Anim. Sci. Pap. Reports.

[62] Tsiplakou E., Zervas G. (2008). Comparative study between sheep and goats on rumenic acid and vaccenic acid in milk fat under the same dietary treatments. Livest. Sci..

[63] Palmquist D.L., Denise Beaulieu A., Barbano D.M. (1993). Feed and Animal Factors Influencing Milk Fat Composition. J. Dairy Sci..

[64] Cossignani L., Giua L., Urbani E., Simonetti M.S., Blasi F. (2014). Fatty acid composition and CLA content in goat milk and cheese samples from Umbrian market. Eur. Food Res. Technol..

[65] Ghaeni M., Ghahfarokhi K.N. (2015). Fatty Acids Profile, Atherogenic (IA) and Thrombogenic (IT) Health Lipid Indices in Leiognathusbindus and Upeneussulphureus. J. Mar. Sci. Res. Dev..

[66] Ulbricht T.L.V., Southgate D.A.T. (1991). Coronary heart disease: Seven dietary factors. The Lancet.

[67] Clark S., García M.B.M. (2017). A 100-Year Review: Advances in goat milk research^1^. J. Dairy Sci..

[68] Salari F., Altomonte I., Ribeiro N.L., Ribeiro M.N., Bozzi R., Martini M. (2016). Effects of season on the quality of Garfagnina goat milk. Ital. J. Anim. Sci..

[69] Fekadu B., Soryal K., Zeng S., Van Hekken D., Bah B., Villaquiran M. (2005). Changes in goat milk composition during lactation and their effect on yield and quality of hard and semi-hard cheeses. Small Rumin. Res..

